# The degradation-promoting roles of deubiquitinases Ubp6 and Ubp3 in cytosolic and ER protein quality control

**DOI:** 10.1371/journal.pone.0232755

**Published:** 2020-05-13

**Authors:** Hongyi Wu, Davis T. W. Ng, Ian Cheong, Paul Matsudaira

**Affiliations:** 1 Temasek Life Sciences Laboratory, National University of Singapore, Singapore, Singapore; 2 Department of Biological Sciences, National University of Singapore, Singapore, Singapore; 3 Mechanobiology Institute, National University of Singapore, Singapore,Singapore; University of Pittsburgh, UNITED STATES

## Abstract

The quality control of intracellular proteins is achieved by degrading misfolded proteins which cannot be refolded by molecular chaperones. In eukaryotes, such degradation is handled primarily by the ubiquitin-proteasome system. However, it remained unclear whether and how protein quality control deploys various deubiquitinases. To address this question, we screened deletions or mutation of the 20 deubiquitinase genes in *Saccharomyces cerevisiae* and discovered that almost half of the mutations slowed the removal of misfolded proteins whereas none of the remaining mutations accelerated this process significantly. Further characterization revealed that Ubp6 maintains the level of free ubiquitin to promote the elimination of misfolded cytosolic proteins, while Ubp3 supports the degradation of misfolded cytosolic and ER luminal proteins by different mechanisms.

## Introduction

Protein quality control (QC) pathways operate in all compartments of eukaryotic cells to eliminate misfolded proteins, the accumulation of which correlates with various age-onset diseases [1–3]. In cytosolic QC (CytoQC), chaperones bind misfolded proteins to inhibit aggregation and assist with refolding [4]. Substrates which fail to refold, such as Ste6*c and ΔssPrA, are degraded by the ubiquitin-proteasome system (UPS) [5–7]. Since many chaperones shuttle between the cytosol and the nucleus, misfolded cytosolic proteins can thus be ferried into the nucleus to be degraded by the nuclear UPS [S1 Fig in S1 File and 8, 9]. Cytosolic aggregates can be re-solubilized by chaperones and degraded via the UPS or directly cleared by autophagy [10]. Similarly, in the endoplasmic reticulum (ER), proteins which misfold in their luminal, transmembrane, or cytosolic domains are engaged by respective ER-associated degradation (ERAD) systems, ERAD-L, ERAD-M and ERAD-C [11], and are retro-translocated into the cytosol for degradation by the UPS [S1 Fig in S1 File and 12]. The model substrates of ERAD include CPY*, Sec61-2 and Ste6* [11, 13–15].

The UPS, which is responsible for degrading the majority of misfolded proteins, consists of the proteasomes and enzymes which catalyze protein ubiquitination, namely the ubiquitin-activating enzyme (E1), -conjugating enzyme (E2) and -ligating enzyme (E3) [16]. Additionally, deubiquitinases (DUbs) such as Ubp6 and Doa4 in *Saccharomyces cerevisiae* (budding yeast) recycle ubiquitin from ubiquitinated proteins [S2 Fig in S1 File and 17, 18–22]. Deubiquitination by various DUbs also regulates different processes such as transcription, translation, signal transduction and vesicle transport [23]. For instance, Ubp3 in yeast deubiquitinates Sec23 to facilitate protein transport by COPII vesicles between ER and Golgi [24, 25].

Although DUbs function in a variety of cellular activities, little is known about the spectrum of DUbs involved in QC or the exact roles of a few DUbs implicated in QC pathways, such as Ubp3 and Ubp6. Ubp3 supports CytoQC under heat stress by suppressing the conjugation of lysine 63 (K63)-linked ubiquitin chains on misfolded proteins and facilitating K48-linkage [26–28], but its function under the physiological temperature or in other QC pathways is unknown [29]. Ubp6 was proposed to delay QC because deleting *UBP6* reduced the steady-state abundance of some proteins [30, 31]. This hypothesis, however, lacks support from direct assays of degradation kinetics [32]. Besides, various studies showed that overexpressing DUbs often impedes QC, but this effect is not observed for DUbs at their physiological concentrations [29, 33–36].

To resolve the roles of DUbs in QC, we screened deletions or mutation of all DUb genes in *S*. *cerevisiae* and quantified their effects on CytoQC and ERAD. We found that half of the deletions decelerate QC whereas the other half have no significant effect. Interestingly, *Δubp6*, which was previously suggested to accelerate QC, delays CytoQC by reducing the level of free ubiquitin, but leaves ERAD unaffected. In contrast, *Δubp3* delays ERAD by compromising the transport between ER and Golgi, and also slows the degradation of a subset of CytoQC substrates by a yet uncharacterized mechanism. These findings demonstrate that the DUbs Ubp6 and Ubp3 support different QC pathways by distinct ways.

## Results

### A reverse genetic screen identified DUbs that support QC degradation

**Fig 1:**
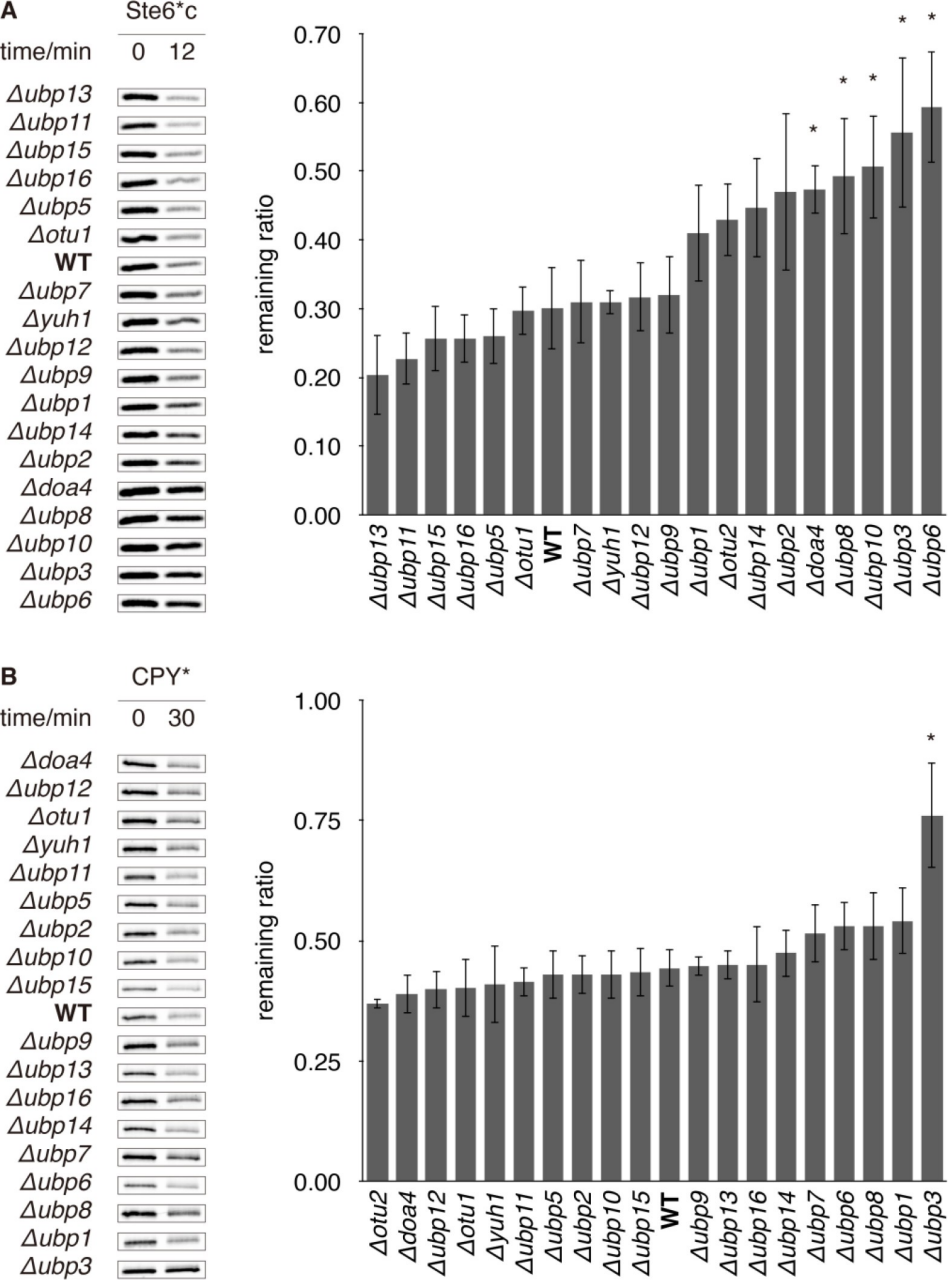
QC is delayed in various DUb deletion strains. (A) Degradation of Ste6*c, a misfolded cytosolic protein, and (B) degradation of CPY*, a misfolded ER protein in WT versus DUb deletion strains. Cells that express Ste6*c or CPY* were pulse-labelled with radioisotopic amino acids and sampled at the indicated time-points. The substrates were then immunoprecipitated, resolved by SDS-PAGE, and exposed to storage phosphor screens. Experiments in this study were performed thrice at 30°C unless otherwise stated. Left: representative gel images. Right: quantification of replicate experiments. The remaining ratio of Ste6*c and CPY* was calculated for each strain as the ratio between the remaining abundance at 12 or 30 min to the initial abundance (at 0 min). Error bars show standard deviations (s.d.). t-tests were performed between mutants and WT. If p-value < 0.05, an asterisk (*) is deposited to the top.

We screened all 20 DUbs in *S*. *cerevisiae* (S2 Fig in S1 File) by measuring the ability of gene deletion or hypomorphic mutation strains to degrade the CytoQC substrate Ste6*c and ERAD substrate CPY*. In wild-type (WT), Ste6*c was rapidly degraded by CytoQC with only 30% of the substrate remaining at 12 min post-labeling (Fig 1A). By contrast, CytoQC was significantly slower in *rpn11*^*S119F*^, *Δubp6*, *Δubp3*, *Δubp8*, *Δubp10* and *Δdoa4* (with over 47% of Ste6*c remaining) and moderately slower in *Δubp2*, *Δubp14*, *Δotu2* and *Δubp1* (with over 41% remaining) (Fig 1A and S3A Fig in S1 File). Degradation was slightly faster in *Δubp13* and *Δubp11* (with 20% and 23% remained) but no further acceleration was observed in the *Δubp11Δubp13* double deletion strain (Fig 1A and S4 Fig in S1 File). The remaining 9 single mutants degraded Ste6*c at WT kinetics (Fig 1A, S3A and S4 Figs in S1 File). As for ERAD, *rpn11*^*S119F*^ and *Δubp3* delayed the degradation of CPY* (with over 76% of CPY* remaining compared to 44% in WT) whereas the remaining mutants, including several which delayed CytoQC (*e*.*g*. *Δubp6*), eliminated CPY* at WT kinetics (Fig 1B and S3B Fig in S1 File). Thus, *rpn11*^*S119F*^, *Δubp6* and *Δubp3* impair CytoQC most severely while *rpn11*^*S119F*^ and *Δubp3* also compromise ERAD. The functions of Ubp6 and Ubp3 in CytoQC and ERAD were further explored.

### Ubp6 promotes CytoQC

**Fig 2:**
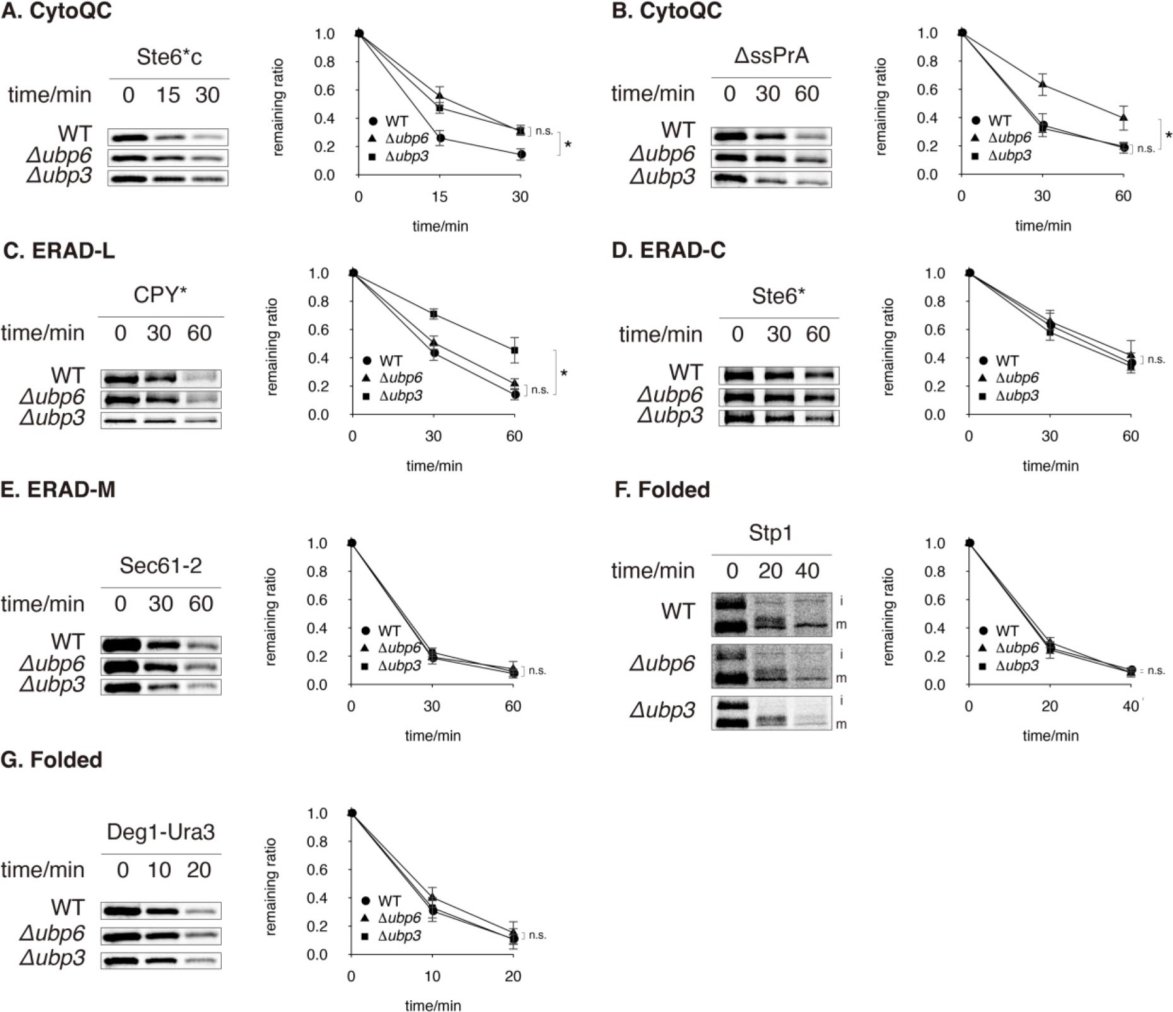
*Δubp6* and *Δubp3* compromise different QC pathways. (A and B) Degradation of cytosolic misfolded proteins Ste6*c and ΔssPrA. (C–E) Degradation of CPY*, Ste6* and Sec61-2, proteins that misfold in the lumen, on the cytosolic surface and in the membrane of ER, respectively. (F and G) Degradation of folded proteins Stp1 and Deg1-Ura3. The uncleaved (immature) and cleaved (mature) forms of Stp1 are respectively indicated as “i” and “m. All substrates were pulsed-labeled and then sampled at the indicated time-points. Their remaining ratios were plotted against time. t-tests were performed for each time-point between different curves. If p-value < 0.05 in at least one t-test, an asterisk (*) is indicated, or otherwise “n.s.” (non-significant) is shown.

Ubp6 is a peripheral subunit of the proteasome which recycles ubiquitin from substrates prior to proteolysis [21, 37–39]. Although *UBP6* deletion had been suggested to enhance QC [30, 31, 40], it in fact compromised the degradation of CytoQC substrate Ste6*c and ΔssPrA (Figs 1A, 2A and 2B). Meanwhile, it did not delay or accelerate the clearance of any ERAD substrate, CPY*, Ste6* or Sec61-2 (Figs 1B and 2C–2E). In no instance was a QC pathway accelerated.

Because other DUbs that promote QC such as Rpn11, Doa4 and Ubp14 (Fig 1A) are also required for degrading folded proteins through non-QC pathways [S3D Fig in S1 File and 20, 22], we examined the role of Ubp6 in the degradation of two folded proteins, Stp1 and Deg1-Ura3. Stp1 is a transcription factor, whose uncleaved cytosolic (immature, i) and cleaved nuclear (mature, m) forms (Fig 2F) are degraded rapidly by the UPS [41]. Deg1-Ura3 is the fusion of Ura3 to the degradation signal (Deg1) of MATα2 and is localized in the cytosol and nucleus [42]. While the elimination of misfolded proteins requires chaperones to maintain solubility or recruit E3, Stp1 and Deg1-Ura3 are degraded in a chaperone-independent manner, which justifies them as folded substrates [4, 43–45]. In *rpn11*^*S119F*^, which served as a control, the degradation of Deg1-Ura3 was significantly decelerated (S3D Fig in S1 File) whereas in *Δubp6* both Deg1-Ura3 and Stp1 were degraded at WT kinetics (Fig 2F and 2G). These results suggest that Ubp6 acts specifically in clearing misfolded proteins by CytoQC.

**Fig 3:**
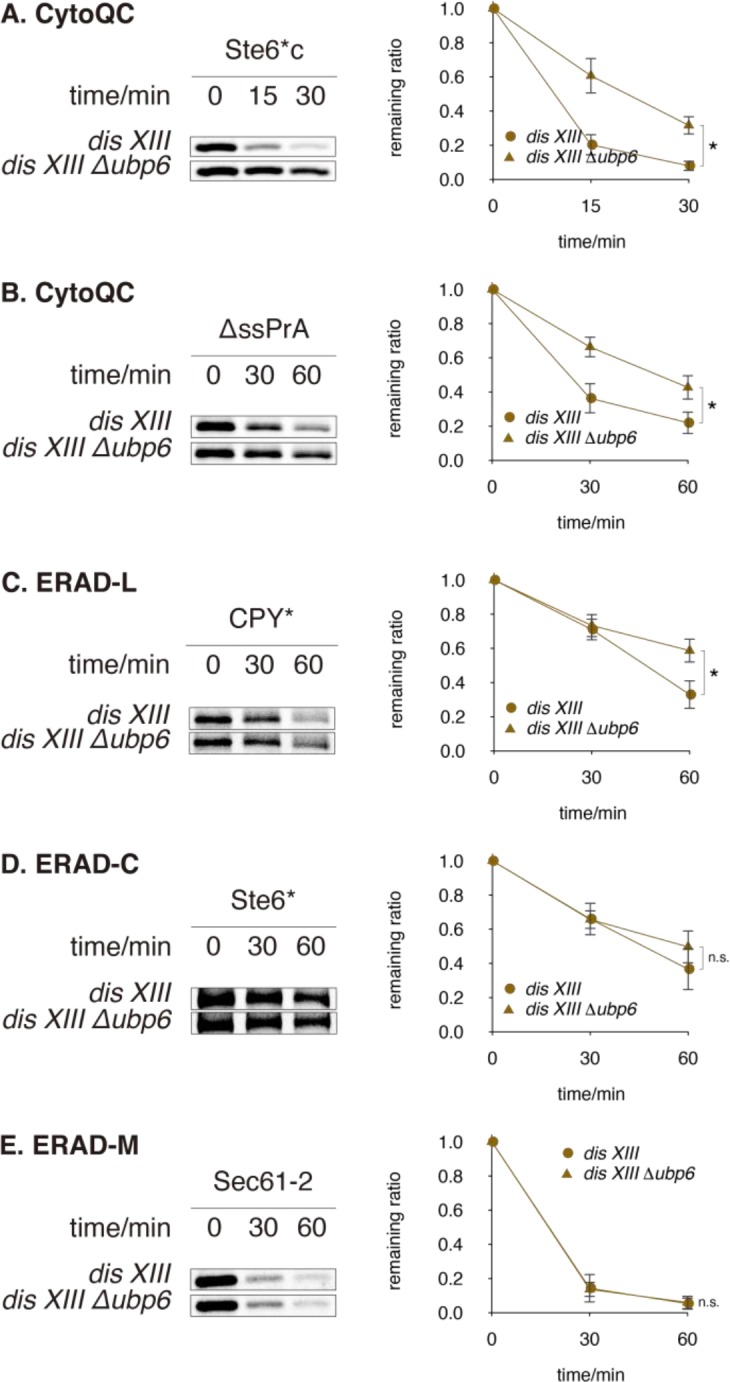
*Δubp6* delays QC in *dis XIII* aneuploid strain. (A and B) Degradation of misfolded cytosolic proteins in *dis XIII* and *dis XIII Δubp6*. (C—E) Degradation of misfolded ER proteins. Substrates were pulsed-chased as in Fig 2.

Ubp6 was originally proposed to delay degradation because in certain aneuploid strains, such as a strain with duplicated *chromosome XIII* (*dis XIII*), its deletion enhances growth [30, 31]. Although our results in haploids have proved otherwise, to test the possibility that Ubp6 functions differently in aneuploid strains, where genes exist in aberrant copy numbers or are expressed differentially [32, 46], we assayed QC in *dis XIII* [30, 31]. As in haploid, *UBP6* deletion significantly compromised CytoQC in *dis XIII* (Fig 3A and 3B), moderately compromised ERAD-L (Fig 3C), and had no effect on ERAD-C or ERAD-M (Fig 3D and 3E). The above evidence proves that Ubp6 is required for efficient CytoQC in both haploid and aneuploid yeast.

### Restoring free ubiquitin abundance in *Δubp6* rescues CytoQC

**Fig 4:**
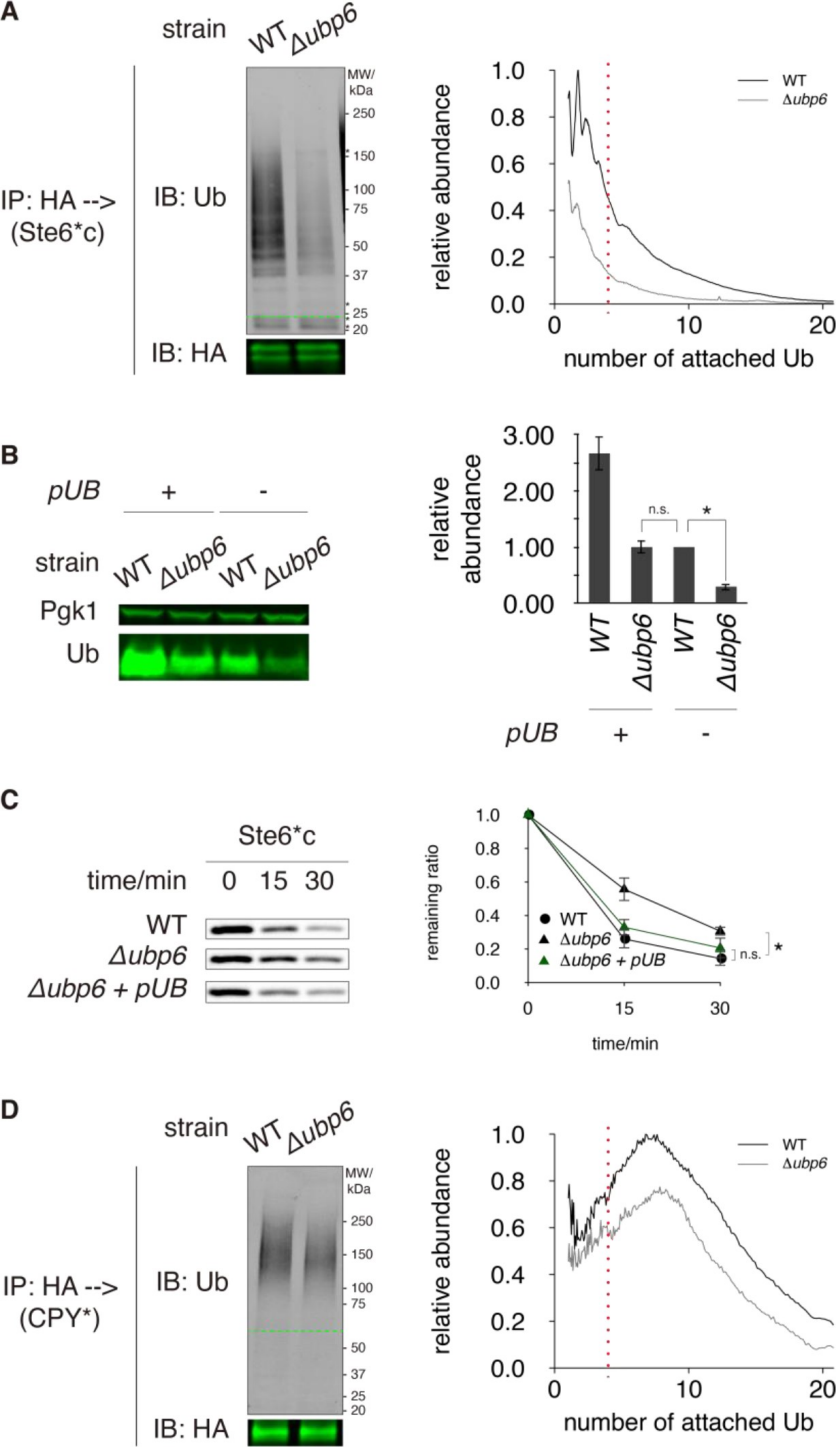
CytoQC is rescued by restoring free ubiquitin abundance in *Δubp6*. (A) Ubiquitination of Ste6*c, a cytosolic misfolded protein. Proteins were extracted under non-reducing condition to preserve unconventional ubiquitination on cysteine residues. Ste6*c was immunoprecipitated (IP) using anti-HA affinity matrix, fractionated by SDS-PAGE (non-reducing) and visualized by immunoblotting against ubiquitin (greyscale) and HA (green). The amount of proteins loaded for IP was normalized based on the level of non-ubiquitinated proteins. Ubiquitinated proteins were observable as smear and ladder in anti-ubiquitin blots. The positions of non-ubiquitinated substrates are indicated by green dashed lines (—). Non-specific bands, which originate from HA affinity matrix, are indicated by asterisks (*). Left: representative blots. Right: quantification of the left. The number of ubiquitin molecules attached to a ubiquitinated species was calculated from the latter’s molecular weight and plotted along the horizontal axis. The abundance of each species, plotted along the vertical axis, was calculated by normalizing the strength of fluorescent signal to the number of ubiquitin moieties and then to the abundance of non-ubiquitinated substrate. The red dashed vertical line indicates where the ubiquitin chain length is 4, the minimum threshold for high-affinity interaction with proteasome. (B) Abundance of free (mono-)ubiquitin in WT and *Δubp6* with or without ubiquitin overexpression (*pUB*). Experiments were performed under non-reducing condition as in (A). Pgk1 was probed as a loading control. (C) Degradation of Ste6*c in *Δubp6* + *pUB*, shown along with degradation in WT and *Δubp6* (without *pUB*). Ste6*c was pulsed-chased as in Fig 2. (D) Ubiquitination of CPY*, a misfolded ER luminal protein. Similar to (A).

Because Ubp6 is a deubiquitinase, we next examined the levels of ubiquitinated CytoQC substrates in WT and *Δubp6*. In WT, the most abundant species of ubiquitinated Ste6*c or ΔssPrA were tagged with di-ubiquitin chains (Fig 4A and S5A Fig in S1 File). As the chain length increased, ubiquitinated substrates decreased in abundance (Fig 4A and S5A Fig in S1 File). In contrast, the abundance of ubiquitinated Ste6*c or ΔssPrA decreased by 50–70% in *Δubp6* and the chain lengths were also shorter (Fig 4A and S5A Fig in S1 File).

Since *Δubp6* exhibited lower ubiquitination levels (Fig 4A and S5A Fig in S1 File), and was known to contain ~ 60% less free ubiquitin [21 and Fig 4B], we tested whether the free ubiquitin pool limits degradation by CytoQC. When the abundance of free ubiquitin in *Δubp6* was restored to WT level or greater (Fig 4B), the degradation of Ste6*c and ΔssPrA recovered to WT kinetics (Fig 4C and S5B Fig in S1 File). In addition, overexpressing ubiquitin in WT almost tripled the abundance of free ubiquitin (Fig 4B) but the kinetics of CytoQC remained the same (S5C Fig in S1 File). These results indicate that when the free ubiquitin pool decreased in *Δubp6* below WT levels, degradation by CytoQC slowed.

If *Δubp6* decelerates CytoQC by reducing the abundance of free ubiquitin, then why is the kinetics of ERAD not affected by this ubiquitin depletion (Fig 2C–2E)? To address this question, we profiled the ubiquitinated species of misfolded ER proteins. The ubiquitin chain lengths of substrates in WT peaked at eight molecules for CPY* (Fig 4D) and at 3 and 9 ubiquitin molecules for Sec61-2 (S5D Fig in S1 File). Interestingly, like CytoQC, the abundance of ubiquitinated CPY* and Sec61-2 decreased by 50–70% *in Δubp6* for species tagged with more than 4 ubiquitin molecules and less so for species tagged with 1–3 ubiquitin molecules (Fig 4D and S5D Fig in S1 File). These profiles showed that although ERAD substrates were degraded at kinetics comparable to WT, they were ubiquitinated to a lesser extent in *Δubp6*, as observed for CytoQC substrates.

### Ubp3 supports CytoQC and ERAD-L

While Ubp3 had been known to support CytoQC under heat stress [28], our genetic screen further revealed that it supports both CytoQC and ERAD at the physiological temperature (30°C) (Fig 1A and 1B). We proceeded to investigate Ubp3’s functions in CytoQC, ERAD and the turnover of folded proteins. *Δubp*3 delayed the clearance of CytoQC substrate Ste6*c (Figs 1A and 2A), but did not influence the clearance of ΔssPrA (Fig 2B). ΔssPrA is distinct from Ste6*c in that it accumulates in the nucleus and is ubiquitinated by the E3 San1 [6–8]. However, Δ2GFP, another San1-dependent and nucleus-localized CytoQC substrate [7, 8], also depended on Ubp3 for clearance (S6A Fig in S1 File). These results show that Ubp3 is required for degrading a subset of cytosolic misfolded substrates but the requirement is not determined by substrate localization or E3 preference.

Deleting *UBP3* also decelerated the clearance of misfolded ER luminal protein CPY* (Figs 1B and 2C) but not the integral ER membrane proteins Ste6* or Sec61-2, which contain a mutation in the cytoplasmic or transmembrane portion respectively (Fig 2D and 2E). To distinguish whether Ubp3 is specifically required by misfolded luminal proteins or any protein with a lesion in the lumen (*i*.*e*. ERAD-L clients), we pulse-chased KWW, a membrane protein with a misfolded luminal domain. The degradation of KWW was delayed in *Δubp3* (S6B Fig in S1 File), demonstrating that the entire branch of ERAD-L is compromised in *Δubp3*.

Similarly, to determine whether *Δubp3* affects the degradation of folded UPS substrates, we pulse-chased Stp1 and Deg1-Ura3. *Δubp3* did not alter the degradation of these two folded substrates (Fig 2F and 2G). Therefore, Ubp3 is specifically required by QC pathways, similar to Ubp6 and distinct from Rpn11, Doa4 and Ubp14.

### Ubp3 uses distinct mechanisms to support CytoQC and ERAD-L

**Fig 5:**
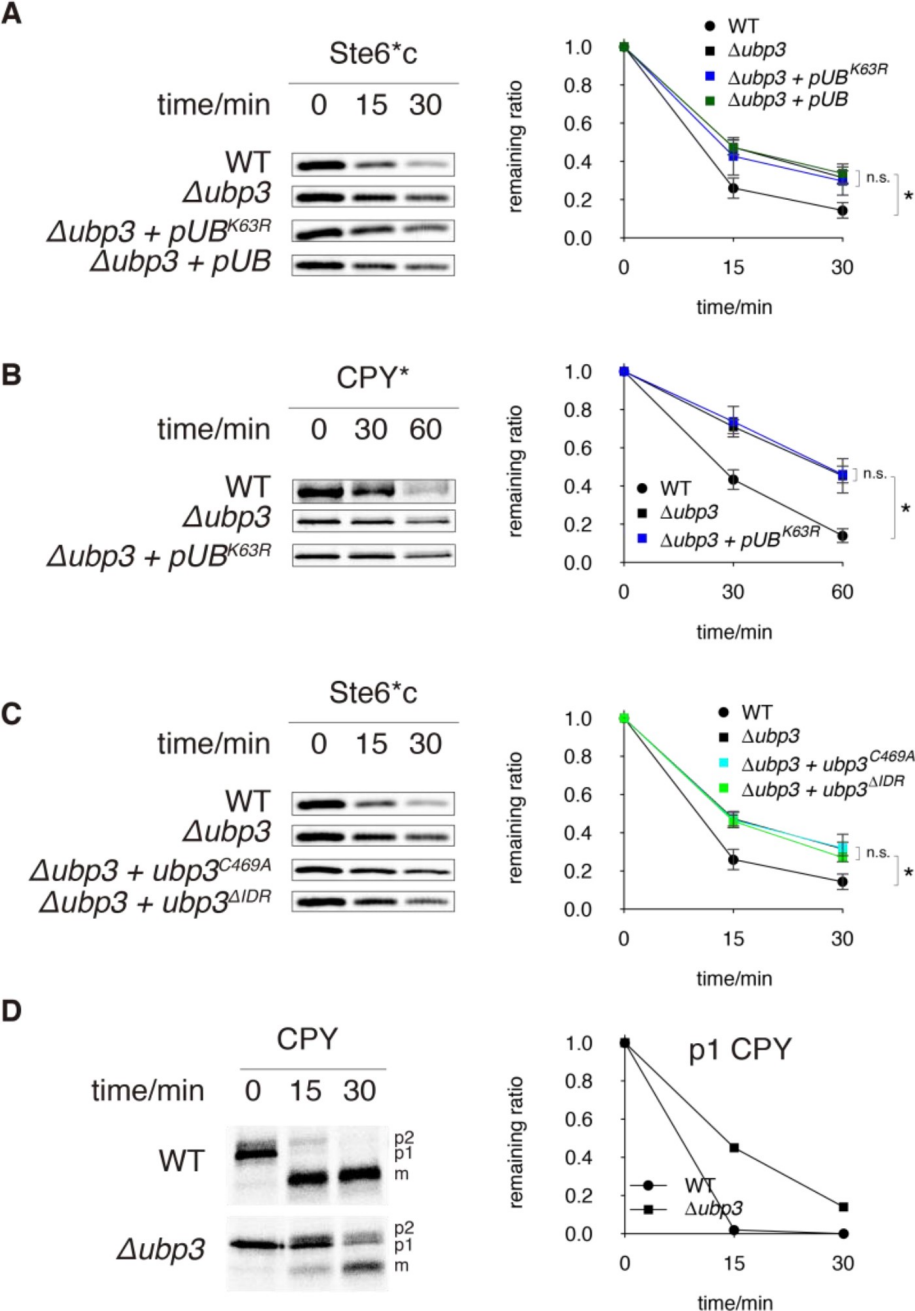
Ubp3 supports QC by distinct mechanisms. (A) Degradation of Ste6*c in *Δubp3* + *pUB*^*K63R*^ and *Δubp3* + *pUB*, assayed by pulse-chase as in Fig 2. The turnover curves in WT and *Δubp3* are displayed as controls. (B) Degradation of CPY* in *Δubp3* + *pUB*^*K63R*^ assayed by pulse-chase, shown along with degradation in WT and *Δubp3*. (C) Degradation of Ste6*c in *Δubp3* + *ubp3*^*C469A*^ and in *Δubp3* + *ubp3*^*ΔIDR*^ assayed by pulse-chase, shown along with degradation in WT and *Δubp3*. (D) Maturation of newly synthesized (WT) CPY, assayed by pulse-chase. p1: ER precursor; p2: precursor that has been transported to and modified by the Golgi; m: mature CPY in vacuole. Graph on the right shows the quantification of p1 CPY at different time-points. Experiment was performed only once.

Under heat stress, Ubp3 promotes CytoQC by exchanging K63- for K48-linkage in ubiquitination so the defect caused by *UBP3* deletion can be surpassed by overexpressing a mutant ubiquitin in which K63 is replaced with arginine (Ub^K63R^) [26–28]. However, at 30°C when Ub^K63R^ was overexpressed in *Δubp3*, CytoQC and ERAD-L remained slow (Fig 5A and 5B), though we used the same construct to rescue protein degradation when *Δubp3* is under heat-stress (S6C Fig in S1 File). This proves that in the absence of heat stress, degradation by QC does not depend on K63-linkage removal by Ubp3. Furthermore, the ubiquitination level of Ste6*c was the same in *Δubp3* and WT (S6D Fig in S1 File) and overexpressing wild-type ubiquitin in *Δubp3* did not rescue CytoQC as in *Δubp6* (Fig 5A). Thus, ubiquitin depletion is not a defect in *Δubp3*.

*Δubp3* also impairs vesicle transport from the ER to Golgi [24 and Fig 5D]. Coincidentally, a delay in ER-to-Golgi transport decelerates ERAD-L but does not affect ERAD-C or -M [47–49], identical to the phenotype of *Δubp3* (Fig 2C–2E). Thus, we reasoned that Ubp3 promotes ERAD-L by facilitating with ER-to-Golgi transport. Furthermore, we investigated if Ubp3 supports CytoQC also by ER-to-Golgi transport, which may affect the nuclear pores (channel for translocating misfolded proteins) or the distribution of CytoQC components such as the proteasomes. However, in *sec12-4*, where ER-to-Golgi transport is impaired (S6E Fig in S1 File), Ste6*c was degraded at WT kinetics (S6F Fig in S1 File). Therefore, ER-to-Golgi transport is not required by CytoQC. Together, our data demonstrates that *Δubp3* delays CytoQC by a novel mechanism.

To investigate this novel mechanism used by Ubp3, we assayed the roles of its C-terminal DUb domain and a largely disordered region (IDR) at the N-terminus. We generated a catalytically-inactive Ubp3 mutant (Ubp3^C469A^) and a mutant without the IDR (Ubp3^ΔIDR^). These mutants were as stable as WT Ubp3 (S6G Fig in S1 File) but were unable to rescue the CytoQC defect in *Δubp3* (Fig 5C), demonstrating that the DUb activity and IDR domain are both required for Ubp3 function.

## Discussion

### A combination of gene deletion and intracellular degradation assay revealed that DUbs promote QC

DUbs are a sizeable class of enzymes with overlaps in function [50, 51]. To bypass their redundancy, the studies of DUbs frequently relied on DUb overexpression. These studies showed that CytoQC in yeast is hindered by overexpressing Ubp1 or Ubp3 [29, 33]. ERAD is hindered by overexpressing the yeast Otu1 or mammalian Usp13, Usp25, *etc*. [34–36]. Nonetheless, the deletion or inhibition of these DUbs either have no effect, such as in *Δubp1* and *Δotu1* [34, 52], or reduces QC efficiency as in the case of *Δubp6*, *Δubp3* and USP13 inhibition [Fig 2 and 28, 35]. Therefore, DUbs may have gained artefactual activities when overproduced. After all, most DUbs recognize substrates by ubiquitin moieties rather than the proteins ubiquitinated [23, 51, 53], so the substrate specificity of these DUbs is determined by whether they localize to the same compartment as substrates or bridged to the substrates by adaptors [24, 54]. Hence, the overproduction of DUbs may impose non-specific interaction between DUbs and substrates. While this method is suitable to study what *deubiquitination* can regulate [55], it does not confirm what a specific DUb does when expressed at the physiological concentration. In this study, we avoided DUb overexpression but used their deletion or hypomorphic mutant strains for analyses.

Another method that infused confusion into understanding DUbs in QC is the use of steady-state protein level as a proxy for degradation rate [30, 56, 57]. However, the abundance of a protein at steady-state is determined by not only degradation but also gene transcription and translation, which DUbs such as Ubp8, Ubp10 and Ubp6 regulate [32, 58, 59]. To avoid such complication, we persistently assayed intracellular degradation by pulse-chase. By coupling gene deletion and mutation with the degradation assay, we revealed that neither CytoQC nor ERAD is accelerated by individual deletion or mutation of the 20 DUb genes in *S*. *cerevisiae*. On the contrary, the main phenotypes observed in these strains were delayed or unaffected degradation of misfolded proteins (Fig 1 and S3A-S3C Fig in S1 File). We further characterized Ubp6 and Ubp3 among the DUbs required for CytoQC or ERAD.

### Ubp6 promotes CytoQC in many potential ways

In the absence of Ubp6, the level of free ubiquitin is dramatically lower than in WT [21 and Fig 4B], and misfolded cytosolic proteins are less ubiquitinated (Fig 4A and S5A Fig in S1 File) and degraded slower (Figs 1A, 2A and 2B). As the delay in CytoQC was fully rescued by restoring free ubiquitin levels (Fig 4C and S5B Fig in S1 File), the most direct interpretation is that CytoQC is compromised by ubiquitin deficiency. Nonetheless, other explanations exist as well.

One alternative explanation lies in the proposed competition between Ubp6 and Rpn11, another DUb in the proteasome which removes ubiquitin chains *en bloc* [60–62]. Deleting *UBP6* may result in the pre-mature deubiquitination of CytoQC substrates by Rpn11 and dissociation from the proteasomes, giving rise to our observed phenotypes.

Another possibility is that proteasomes become less active in *Δubp6*. According to structural biology analyses, deubiquitination by Ubp6 “lubricates” the translocation of substrates into the proteasome chamber, where proteolysis occurs [60–62]. In addition, as a peripheral subunit of the proteasome, Ubp6 can induce conformational change in the proteasome to favor the degradation of certain substrates [38, 39]. If in *Δubp6* the translocation of CytoQC substrates or change in proteasome conformation is hindered, then degradation becomes slowed and ubiquitinated substrates would accumulate. Nevertheless, if deleting *UBP6* simultaneously delays the ubiquitination of CytoQC substrates by ubiquitin depletion, the abundance of ubiquitinated substrates could still decrease, consistent with what we observed. Similarly, it is possible that certain steps in the pathway is accelerated in *Δubp6* despite an overall delay in CytoQC and still not faster when ubiquitin level is restored (Fig 4C). To reveal if any step in CytoQC is enhanced by *UBP6* deletion will require other techniques such as single-molecule tracking to follow the fates of different populations of misfolded proteins.

### Ubp6 is not required by ERAD or degradation of folded proteins

In contrast to CytoQC, ERAD was not delayed in *Δubp6* (Fig 2C–2E) even though the substrates were less ubiquitinated (Fig 4D and S5D Fig in S1 File). This observation supports the notion that the rate-limiting step in ERAD is not ubiquitination but retro-translocation or extraction of proteins from the ER membrane [63–66].

Similarly, the degradation of folded proteins does not require Ubp6. In *Δubp6*, the short-lived folded proteins Stp1-HA and Deg1-Ura3 were degraded at WT rates (Fig 2F and 2G). Whether the ubiquitination of these folded proteins is affected by *UBP6* deletion remains unclear as we were unable to quantify their ubiquitinated species due to low abundance. Our result is consistent with a previous report that various long-lived proteins in *Δubp6* displayed WT degradation rates [32]. Together, we conclude that Ubp6 is not required for the degradation of ERAD or folded substrates but is specific for misfolded cytosolic proteins.

### Ubp3 supports QC at normal temperature

Proteins under heat-stress are decorated with ubiquitin chains of higher content of K63-linkage, as Rsp5 becomes a major E3 that catalyzes ubiquitination [26–28]. Ubp3 was found to associate with Rsp5 at higher temperature to exchange K63- for K48- linkage. Deletion of *UBP3* under the same condition results in slower degradation and accumulation of protein aggregates [28, 29]. Our study further revealed that at the physiological temperature, Ubp3 still promotes CytoQC (Figs 1A, 2A and S6A Fig in S1 File) even though Rsp5 is no longer involved [26–28]. Interestingly, Ubp3 is required by only a subset of CytoQC substrates, indicating the existence of two CytoQC branches of distinct Ubp3-reliance. Moreover, the promotion of CytoQC by Ubp3 is unrelated to the remodeling of K63- into K48- linkage (Fig 5A) or to the level of substrate ubiquitination (S6D Fig in S1 File). Currently, we hypothesize that Ubp3 trims ubiquitin chains of certain forked topology, which has been reported to inhibit degradation [67]. To test this hypothesis, mass-spectrometry must be employed to determine and compare the topology of ubiquitin chains installed on CytoQC substrates in *Δubp3* and WT.

At 30°C, we also discovered that Ubp3 supports ERAD-L (Figs 1B and 2C). Ubp3 utilizes Bre5 as a cofactor to recognize and deubiquitinate Sec23 [24, 25]. This process is required for ER-to-Golgi transport (Fig 5C), which in turn is implicated in ERAD-L [47, 48]. However, it is confirmed that ERAD-L substrates *per se* need not undergo ER-to-Golgi transport before degradation [48, 49], so the exact mechanism of how ER-to-Golgi transport maintains ERAD-L remains to be unveiled [48].

### The localization and domain organization of DUbs determine their functions in QC

As presented in this article, Ubp6 and Ubp3 use different mechanisms to promote degradation of distinct sets of misfolded proteins (Figs 2, 4 and 5). In addition, three other hits in our genetic screen, namely Rpn11, Doa4 and Ubp14, also display different roles in CytoQC and ERAD [Fig 1, S3 Fig in S1 File and 20, 22]. These differences in DUb functions arise from their diverse subcellular localization and domain organization. Rpn11 is situated at the proteasome but closer to the entry of the catalytic chamber than Ubp6 [38, 39]. Therefore, the *rpn11*^*S119F*^ hypomorphic mutation likely causes a defect in substrate translocation into the proteasome chamber [61, 62]. This defect slowed both CytoQC and ERAD and is not rescued by ubiquitin overexpression (S3A-S3E Fig in S1 File). Doa4 is physically associated with endosomes by its N-terminal segment [54 and S2 Fig in S1 File] and its deletion results in accumulation of small ubiquitin conjugates [22, 68]. Ubp14 contains zinc finger (ZF) domains which recognize the C-terminus of unanchored polyubiquitin chains to stimulate the DUb activity [20 and S2 Fig in S1 File, 69]. Because neither *Δdoa4* nor *Δubp14* causes ubiquitin deficiency as severe as *Δubp6*, the accumulation of small ubiquitin conjugates or unanchored polyubiquitin in these strains could be responsible for the decelerated CytoQC [Fig 1A and 20, 22, 54, 68]. However, why they leave ERAD unaffected remains to be explored (Fig 1B).

In conclusion, it is now clear that deletions of individual DUbs do not accelerate QC in *S*. *cerevisiae*. On the contrary, DUbs such as Ubp6 and Ubp3 promote different QC pathways by distinct mechanisms including ubiquitin recycling and the maintenance of vesicle transport. Further investigation into these diverse mechanisms will aid in our understanding of how CytoQC and ERAD are organized to efficiently clear aberrant proteins.

## Materials and methods

### Euploid yeast strains and culture

Strains used in the gene deletion screen were derived from *S*. *cerevisiae BY4742* (*s288c his3Δ1 leu2Δ0 lys2Δ0 ura3Δ0*, YSC1049 from Dharmacon). *rpn11*^*S119F*^ and *sec12-4* were derived from *W303* (*leu2-3*,*112 trp1-1 can1-100 ura3-1 ade2-1 his3-11*,*15*). Others were derived from *RLY2626* (*s228c ura3 his3 trp1 leu2 LYS2*) [46]. Gene deletion strains were generated by PCR-based gene knock-out [70, 71]. Euploid yeast strains were maintained by typical methods [72], and for experiments, cultured in synthetic media at 30°C to mid-exponential phase (A_600_ ≈ 0.7). A list of *S*. *cerevisiae* strains used in this study can be found in Table 1.

**Table 1:** Yeast strains. *kanMX* is a gene cassette that enables yeast to grow with G418 (geneticin).

identifier	background	genotype	source	comment
YY286	*BY4742*	WT *(his3Δ1 leu2Δ0 lys2Δ0 ura3Δ0) MATα*	Dharmacon	
YY503	*BY4742*	*Δubp1*::*kanΜΧ*	Dharmacon	
YY504	*BY4742*	*Δubp2*::*kanΜΧ*	Dharmacon	
YY505	*BY4742*	*Δubp3*::*kanΜΧ*	Dharmacon	
YY506	*BY4742*	*Δdoa4*::*kanΜΧ*	Dharmacon	
YY507	*BY4742*	*Δubp5*::*kanΜΧ*	Dharmacon	
YY473	*BY4742*	*Δubp6*::*kanΜΧ*	Dharmacon	
YY508	*BY4742*	*Δubp7*::*kanΜΧ*	Dharmacon	
YY509	*BY4742*	*Δubp8*::*kanΜΧ*	Dharmacon	
YY510	*BY4742*	*Δubp9*::*kanΜΧ*	Dharmacon	
YY1265	*BY4742*	*Δubp10*::*KanMX*	this study	
YY511	*BY4742*	*Δubp11*::*kanΜΧ*	Dharmacon	
YY512	*BY4742*	*Δubp12*::*kanΜΧ*	Dharmacon	
YY513	*BY4742*	*Δubp13*::*kanΜΧ*	Dharmacon	
YY514	*BY4742*	*Δubp14*::*kanΜΧ*	Dharmacon	
YY515	*BY4742*	*Δubp15*::*kanΜΧ*	Dharmacon	
YY516	*BY4742*	*Δubp16*::*kanΜΧ*	Dharmacon	
YY517	*BY4742*	*Δyuh1*::*kanΜΧ*	Dharmacon	
YY752	*BY4742*	*Δotu1*::*kanΜΧ*	Dharmacon	
YY1566	*BY4742*	*Δotu2*::*kanΜΧ*	Dharmacon	
YY1322	*BY4742*	*Δubp11*::*kanΜΧ Δubp13*::*LEU2*	this study	
YY282	*W303*	*WT (leu2-3*,*112 trp1-1 can1-100 ura3-1 ade2-1 his3-11*,*15)*	Davis Ng lab stock	
YY329	*W303*	*rpn11*^*S119F*^	our submitted manuscript	
YY688	*RLY2626*	WT *(ura3 his3 trp1 leu2 LYS2) MATa*	Pavelka *et al*., 2010	
YY738	*RLY2626*	*MATa Δubp6*::*LEU2*	this study	
YY1418	*RLY2626*	*MATa Δubp3*::*kanMX*	this study	
YY688	*RLY2626*	WT *MATα*	this study	*MAT* switched from YY688
YY738	*RLY2626*	*MATα Δubp6*::*LEU2*	this study	*MAT* switched from YY738
YY1491	*RLY2626*	*MATα Δubp3*::*kanMX*	this study	
YY882	*RLY2626*	*dis XIII MATα*	Pavelka *et al*., 2010	
YY905	*RLY2626*	*dis XIII MATα Δubp6*::*LEU2*	this study	
YY1479	*W303*	*sec12-4*	Vashist *et al*., 2001	

### Retrieval and generation of DUb mutants

We retrieved the deletion strains of non-essential DUbs, except for Ubp10, from a deletion library sold by Dharmacon. Their identities were re-confirmed by PCR genotyping. *Δubp10* was not provided by the deletion library so we generated this mutant on our own. For Rpn11, which is essential for cell growth, we acquired from a genetic selection for CytoQC components an *rpn11*^*S119F*^ mutant, which is reduced in its Zn^2+^-coordinating ability required for deubiquitination by this metalloprotease [73, 74 and our submitted manuscript].

### Aneuploid yeast strains and culture

*dis XIII* aneuploid strain is a kind gift from G. Rancati and R. Li [46]. This strain is derived from *RLY2626*. Aneuploid strains were always maintained at 25°C. The ploidy of all aneuploid strains was verified by qPCR karyotyping (below). When aneuploid cells were cultured at 30°C for pulse-chase (Fig 3), an aliquot of the same culture was also karyotyped.

### qPCR karyotyping

*S*. *cerevisiae* cells of the exponential phase were diluted to A_600_ = 0.3. Of the normalized culture, 300 μL was taken and cells were washed with phosphate buffered saline (PBS, pH = 7.5). Then cell walls were digested by 14 mg/mL of zymolyase 20T (US Biologicals Z1000) in 21.5 μL of PBS plus 2.3 mM of DTT. Afterwards, the genomic DNA was released by boiling at 100°C for 5 min. 0.5 μL of the cell lysate was used in qPCR, performed using reagents and protocol from the QuantiNova SYBR Green PCR Kit (Qiagen 204141). Primers for karyotyping were published previously [46]. The variation of *chr XIII* copy number was within ± 0.2 at the population level.

### Substrates and plasmids

Substrates of the UPS examined in this study (S1 Fig in S1 File) were hosted on centromeric plasmids. Among them, KWW is HA-tagged at the C-terminus of its KHN domain and Deg1-Ura3 is not tagged. Other proteins are HA-tagged at their C-termini. A list of plasmids used in this study are shown in Table 2. All insertions on plasmids have been validated by sequencing.

**Table 2:** Plasmids. ***pRS313*, *pRS314* and *YCp50* are centromeric vectors while *pRS424* is a 2μ vector [75, 76].** *TDH3* and *PRC1* promoters are strong and moderate constitutive promoters, respectively. All plasmids harbor *ACT1* terminator downstream the genes expressed. Plasmid maps and sequences are available on request.

identifier	vector	promoter	encoded protein	tag	source	comment
pRP22	*pRS313*	*TDH3*	Ste6*c	C-terminal HA	Prasad *et al*., 2012	
pRP42	*pRS313*	*TDH3*	ΔssPrA	C-terminal HA	Prasad *et al*., 2010	
pY129	*pRS313*	*PRC1*	CPY*	C-terminal HA	this study	
pY203	*pRS313*	*TDH3*	Ste6*	C-terminal HA	this study	also expressed in *MATα* strains
pY204	*pRS313*	*SEC61*	Sec61-2	C-terminal HA	this study	
pY194	*pRS313*	*STP1*	STP1	C-terminal HA	this study	
pY192	*pRS313*	*MATALPHA2*	Deg1-Ura3	none	this study	
pY109	*pRS314*	*TDH3*	Ub	none	this study	alias: *pUB*
pY225	*pRS424*	*PRC1*	Ub^K63R^	none	this study	alias: *pUB*^K63R^
pRP44	*pRS313*	*TDH3*	Δ2GFP	C-terminal HA	Prasad *et al*., 2010	
pSM101	*YCp50*	*PRC1*	KWW	3xHA at the C-terminus of KHN domain	Vashist and Ng, 2004	
pY237	*pRS316*	*UBP3*	Ubp3	C-terminal FLAG	this study	
pY239	*pRS316*	*UBP3*	Ubp3^C469A^	C-terminal FLAG	this study	
pY241	*pRS316*	*UBP3*	Ubp3^ΔIDR^	C-terminal FLAG	this study	

### Antibodies

To immunoprecipitate HA-tagged proteins and to detect HA-tagged proteins in immunoblotting, the anti-HA monoclonal mouse antibody HA.11 (BioLegend 901501) was routinely used. Other antibodies, antisera and affinity matrix used in this study are: anti-Ura3 rabbit serum (raised in lab), anti-Ub monoclonal mouse antibody Ubi-1 (invitrogen 13–1600), anti-Pgk1 monoclonal mouse antibody (Invitrogen 459250), anti-FLAG mouse monoclonal antibody M2 (Sigma F1804), anti-Sec61 rabbit serum (kind gift from P. Walter), anti-CPY rabbit serum (kind gift from R. Gilmore), and anti-HA affinity matrix (Roche 11815016001).

### Metabolic ^35^S labelling and pulse-chase

*S*. *cerevisiae* cells of the mid-exponential phase were concentrated 5 times in fresh media and allowed 30 min to adapt. Cells were then labelled by adding the EXPRE^35^S^35^S Protein Labeling Mix (PerkinElmer EasyTagTM NEG772) at a ratio of 9 μL per mL of the concentrated culture. After 5 or 10 min, pulse-labelling was quenched by adding 12.5 μL of chase media (200 mM methionine, 200 mM cysteine) for each mL of the culture. 1 mL of the culture was then aliquoted at different time-points post-labelling and all cellular activities in the aliquot were killed immediately by adding trichloroacetic acid (TCA) to 10% (v/v). After protein extraction and the immunoprecipitation of substrates (see below), samples were fractionated by SDS-PAGE. Gels were dried and exposed to storage phosphor screens (Kodak SO230 or Fuji BAS-IP SR 2025). Finally, the screens were scanned by a Typhoon 9200 Scanner or an IP Biomolecular Imager (GE Healthcare) and analyzed in ImageQuant TL.

### Protein extraction

Cells killed by 10% TCA were subsequently lysed by bead beating. Then, proteins were precipitated by centrifugation (> 18000 g, 15 min at 4°C) and for each mL of yeast culture in exponential phase, dissolved in 16–35 μL of TCA resuspension buffer (3% SDS [w/v], 100 mM Tris pH = 9.0, 3 mM DTT) by boiling at 100°C and vortexing. For ubiquitination assay (below), DTT was omitted from TCA resuspension buffer to extract proteins under non-reducing condition.

### Immunoprecipitation (IP)

50 μL of protein extract (in TCA resuspension buffer) was mixed with 700 μL of IP solution II (20 mM Tris pH = 7.5, 150 mM NaCl, 1% [w/v] Triton X-100, 0.02% [w/v] NaN_3_), 6 μL of 100 mM PMSF, 1 μL of protease inhibitor cocktail (Sigma P8215) and 1–5 μL of antibody solution. The mixture was incubated for 1 h under 4°C. After removing insoluble materials by centrifugation (> 18000 g, 20 min at 4°C), the supernatant was mixed with 30 μL of protein A Sepharose (Sigma P3391) and rotated for 2 h at 4°C. Protein A beads were then washed 3 times with IP solution I (IP solution II + 0.2% [w/v] SDS) and 2 times with PBS. Finally, proteins were eluted into ~ 25 μL of PBS plus 10 μL of 4x Laemmli buffer (125 mM Tris-HCl, pH = 6.8, 4% [w/v] SDS, 50% [v/v] glycerol, 0.2 mg/mL bromophenol blue, 5% [v/v] β-mercaptoethanol) by boiling at 100°C and vortexing.

### Immunoblotting (IB)

Nitrocellulose membranes (BIO-RAD 1620213 or 1704159) were used for the electroblotting of proteasomal substrates. PVDF (BIO-RAD 1704156) was used for the blotting of free Ub and was autoclaved in water after blotting [77]. After blocking in Odyssey Blocking Buffer (PBS, LI-COR 927), membranes were incubated sequentially with primary and secondary antibodies in Odyssey Blocking Buffer mixed with equal volume of PBS and Tween 20 at 0.1% (v/v). After each incubation, membranes were washed in PBS plus 0.1% (v/v) of Tween 20. Tween 20 was removed by rinsing in PBS before detecting the fluorescence of secondary antibodies using a LI-COR Odyssey Classic Scanner. The fluorescence of protein bands was quantified by Odyssey Application Software while the ubiquitination profiles were quantified in ImageQuant TL by 1D Gel Analysis.

### Ubiquitination assay

Proteins were extracted under non-reducing condition to preserve unconventional ubiquitination on cysteine residues [66]. Up to 85 μL of the protein extract, normalized to contain equal amounts of un-modified substrates, was mixed with 50 μL of anti-HA affinity matrix, 1200 μL of IP solution II, 1.8 μL of PIC and 10.5 μL of PMSF to immunoprecipitate HA-tagged proteins. Products of IP were fractionated by non-reducing SDS-PAGE and electroblotted (4°C overnight) onto nitrocellulose membranes. The blots were autoclaved to better expose the antigen [77] and the ubiquitinated species were detected by immunoblotting against ubiquitin (weak fluorescent signal). The non-ubiquitinated species was subsequently visualized by blotting against HA (strong fluorescent signal).

### Scintillation counting of radioactive samples

2.5–10 μL of protein samples in TCA resuspension buffer was mixed with 4 mL of scintillation cocktail (RPI Bio-Safe NA 111198), which pre-mixed with 0.1 volume of isopropanol to suppress precipitation proteins precipitation. Scintillation was measured on a PerkinElmer Tri-Carb 4810TR liquid scintillation analyzer.

### Cycloheximide (CHX)-chase

CHX was added into yeast culture to a final concentration of 200 μg/mL to inhibit protein translation. After certain periods of treatment, equal amounts (4.5 mL) of yeast culture was removed and mixed with TCA (final concentration = 10% [v/v]). Proteins were then extracted into TCA resuspension buffer and mixed with proper amounts of 4x Laemmli buffer. After heating at 100°C for 10 min, samples were loaded for SDS-PAGE. Substrates were detected by immunoblotting and quantified by Odyssey Application Software.

## Supporting information

S1 File (DOCX)

## Abbreviations

chr: chromosome
CPY: carboxypeptidase Y
CytoQC: cytosolic (protein) quality control
dis: disomic
DTT: dithiothreitol
DUb: deubiquitinase
ER: endoplasmic reticulum
ERAD: ER-associated protein degradation
GFP: green fluorescent protein
HA: hemagglutinin tag
IP: immunoprecipitation
PAGE: polyacrylamide gel electrophoresis
PBS: phosphate-buffered saline
PIC: protease inhibitor cocktail
PMSF: phenylmethylsulfonyl fluoride
PrA: proteinase A
pUB: *pRS313* centromeric plasmid expressing ubiquitin from *TDH3* promoter
pUB^K63R^: *pRS424* 2μ plasmid expressing ubiquitin from *PRC1* promoter
QC: (protein) quality control
qPCR: quantitative PCR
SDS: sodium dodecyl sulfate
ss: signal sequence
TCA: trichloroacetic acid
Ub: ubiquitin
UPS: ubiquitin-proteasome system
WT: wild-type
