# Supplementary material for: The degradation-promoting roles of deubiquitinases Ubp6 and Ubp3 in cytosolic and ER protein quality control
Source: PLoS One. 2020 May 13;15(5):e0232755. doi: 10.1371/journal.pone.0232755 (PMC7219781; doi:10.1371/journal.pone.0232755)
Supplement: S1 File — (DOCX) [file pone.0232755.s001.docx]

Supplemental Information

**Manuscript title: The Degradation-promoting Roles of Deubiquitinases Ubp6 and Ubp3 in Cytosolic and ER Protein Quality Control**

Hongyi Wu ^1, 2, *^, Davis T.W. Ng ^1^, Ian Cheong ^1, 2, *^ and Paul Matsudaira ^2, 3, *^

^1^ Temasek Life Sciences Laboratory, 1 Research Link, National University of Singapore, Singapore 117604

^2^ Department of Biological Sciences, National University of Singapore, Block S3 #05-01, 16 Science Drive 4, Singapore 117558

^3^ Mechanobiology Institute, National University of Singapore, T-Lab #10-01, 5A Engineering Drive 1, Singapore 117411

^*^ To whom correspondence should be addressed.

Hongyi Wu ([mbiwh@nus.edu.sg](mailto:mbiwh@nus.edu.sg). Current address: Mechanobiology Institute, National University of Singapore) or Paul Matsudaira ([dbsmpt@nus.edu.sg](mailto:dbsmpt@nus.edu.sg)) or Ian Cheong ([ian@tll.org.sg](mailto:ian@tll.org.sg))

# Supplemental figure legends

## Figure S1

Substrates used in this study. These substrates were selected to cover the canonical pathways of QC and the degradation of folded proteins via the UPS. (**A and B**) CytoQC substrates: Ste6*c and ΔssPrA [1, 2]. (**C - E and H**) ERAD substrates: (C and H) ERAD-L substrates CPY* and KWW; (D) ERAD-C substrate Ste6*; (E) ERAD-M substrate Sec61-2 [3-6]. (**F and G**) Folded UPS substrate Stp1 and Deg1-Ura3 [7-9]. For each substrate, a schematic diagram is provided. Black vertical cylinder ( **|** ): transmembrane helix; black curve ( ∩ ): loop region; red asterisk (*): mutation/misfolded site; green oval (**•**): folded domain; black solid arrow line (🡪): translocation of substrate. Diagrams are not to scale.

## Figure S2

List of yeast DUbs. The catalytic domains of these DUbs fall into 4 families: ubiquitin-specific protease (USP), ubiquitin C-terminal hydrolase (UCH), ovarian tumor (OTU) and JAB1/MPN/Mov34 metalloenzyme (JAMM). Other domains/regions found in DUbs are: transmembrane (TM), repeats (RPT), disordered region (IDR), rhodanese homology domain (rhodanese), ubiquitin-like (UBL) domain, zinc finger (ZF), ubiquitin-associated (UBA) domain and signal sequence (ss). NLS: Nuclear Localization Signal. The domain structures of DUbs were identified in Pfam and rendered in DoMosaics [10, 11]. NLS was predicted by NLS Mapper using default settings [12].

## Figure S3

*rpn11* mutation at its active site (*rpn11^S119F^*) impairs all degradation pathways via the UPS. (**A**) Degradation of Ste6*c in *RPN11* and *rpn11^S119F^* (*W303* background) in the absence or presence of ubiquitin overexpression. (**B - D**) Degradation of CPY*, Sec61-2 and Deg1-Ura3 in *RPN11* and *rpn11^S119F^*. Substrates in (A - D) were pulsed-chased only once in *rpn11^S119F^*. (**E**) Free ubiquitin abundance in *RPN11* and *rpn11^S119F^*, assayed as in Figure 4B.

## Figure S4

Degradation of Ste6*c in WT, *Δubp11*, *Δubp13* and *Δubp11Δubp13*, assayed by pulse-chase as in Figure 2.

## Figure S5

Supplemental to Ubp6 mechanism. (**A**) Ubiquitination of ΔssPrA, assayed and presented as in Figure 4A. (**B**) Degradation of ΔssPrA in *Δubp6* + *pUB*, shown along with degradation in WT and *Δubp6* (without *pUB*). (**C**) Degradation of Ste6*c in WT and WT + *pUB*. (**D**) Ubiquitination of Sec61-2, a misfolded ER membrane protein (with lesion in membrane). Products of immunoprecipitation from strains without harboring substrates were run in adjacent lanes as control. Empty circle: vector control; filled circle: Sec61-2 expressed.

## Figure S6

Supplemental to Ubp3 mechanism. (**A and B**) Degradation of Δ2GFP and KWW in *Δubp3* versus WT, assayed by pulse-chase as in Figure 2. (**C**) Degradation of newly synthesized proteins under heat shock. Cells were cultured at 25 ℃ and shifted to 37 ℃ for 30 min before they were pulse-chased as in Figure 5A. The abundance of nascent proteins was quantified by TCA-precipitable (scintillation) counts. The ratios between degraded proteins and initially labelled proteins are plotted against time. (**D**) Ubiquitination of Ste6*c in *Δubp3* versus WT, assayed as in Figure 4A. (**E**) Maturation of CPY in *sec12-4* (of W303 background) and isogenic WT (*SEC12*). Cells were cultured and pulse-chased at 30℃. (**F**) Degradation of Ste6*c in *sec12-4* (of W303 background) and isogenic WT (*SEC12*). Cells were cultured and pulse-chased at 30℃, the semi-permissive temperature of *sec12-4* (panel E). (**G**) Cycloheximide (CHX) chase of Ubp3 and variants. CHX was added to WT cells that express Ubp3, Ubp3^C469A^ or Ubp3^ΔIDR^ (FLAG-tagged) to inhibit protein translation. Samples were then taken at the indicated time and immunoblotted against FLAG. Sec61 was probed as a loading control. Experiments in (B, D, E and G) were performed only once.

## Figure S7

Uncropped images.

# Supplemental figures

## Figure S1


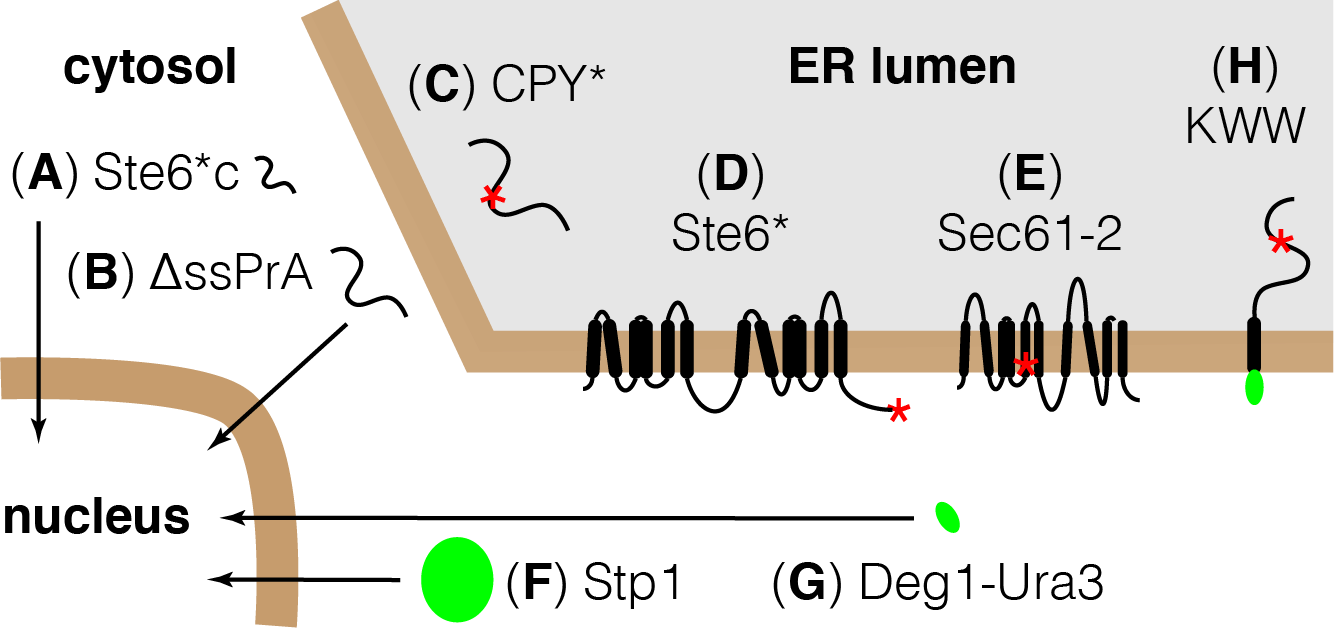


## Figure S2


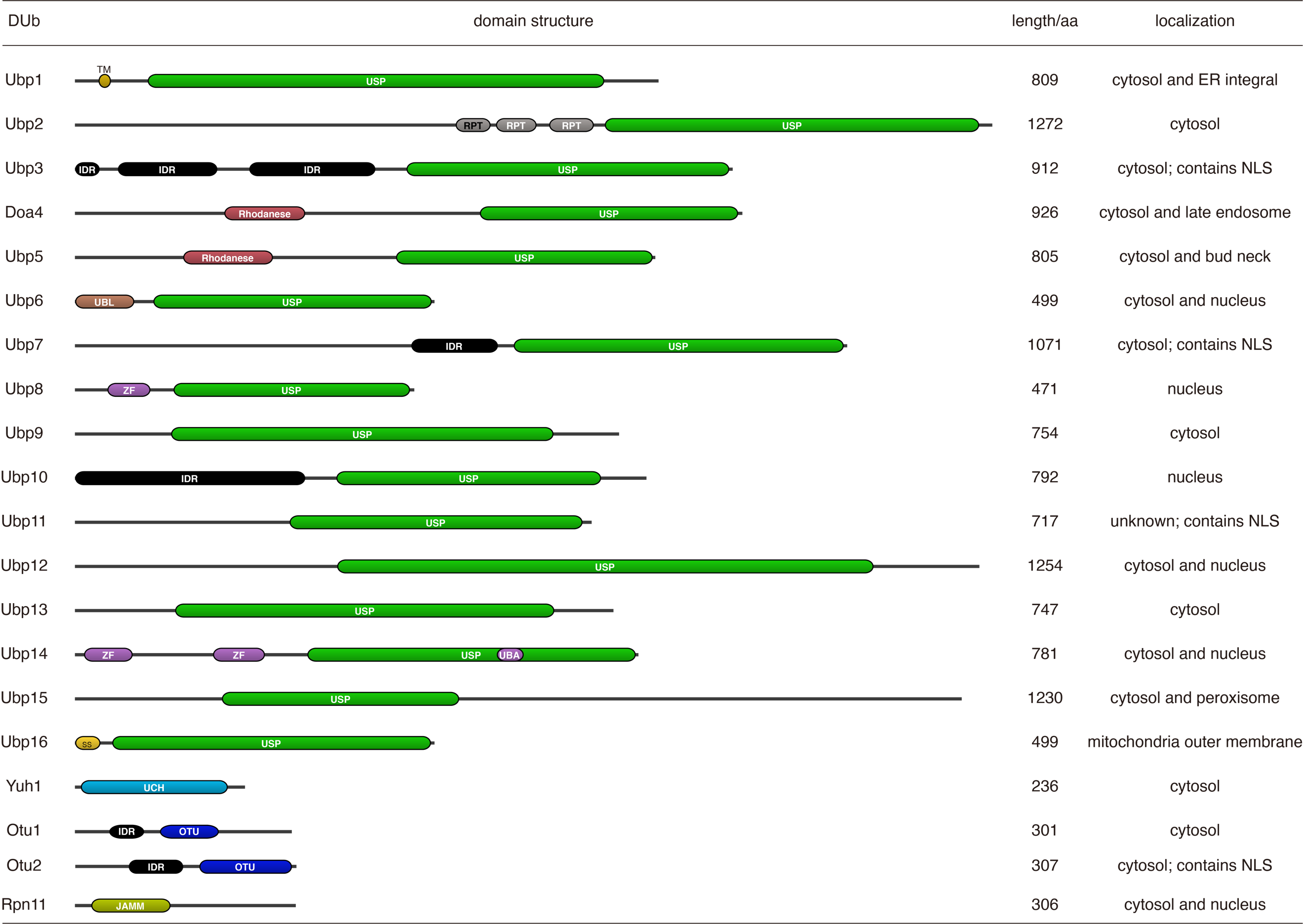


## Figure S3


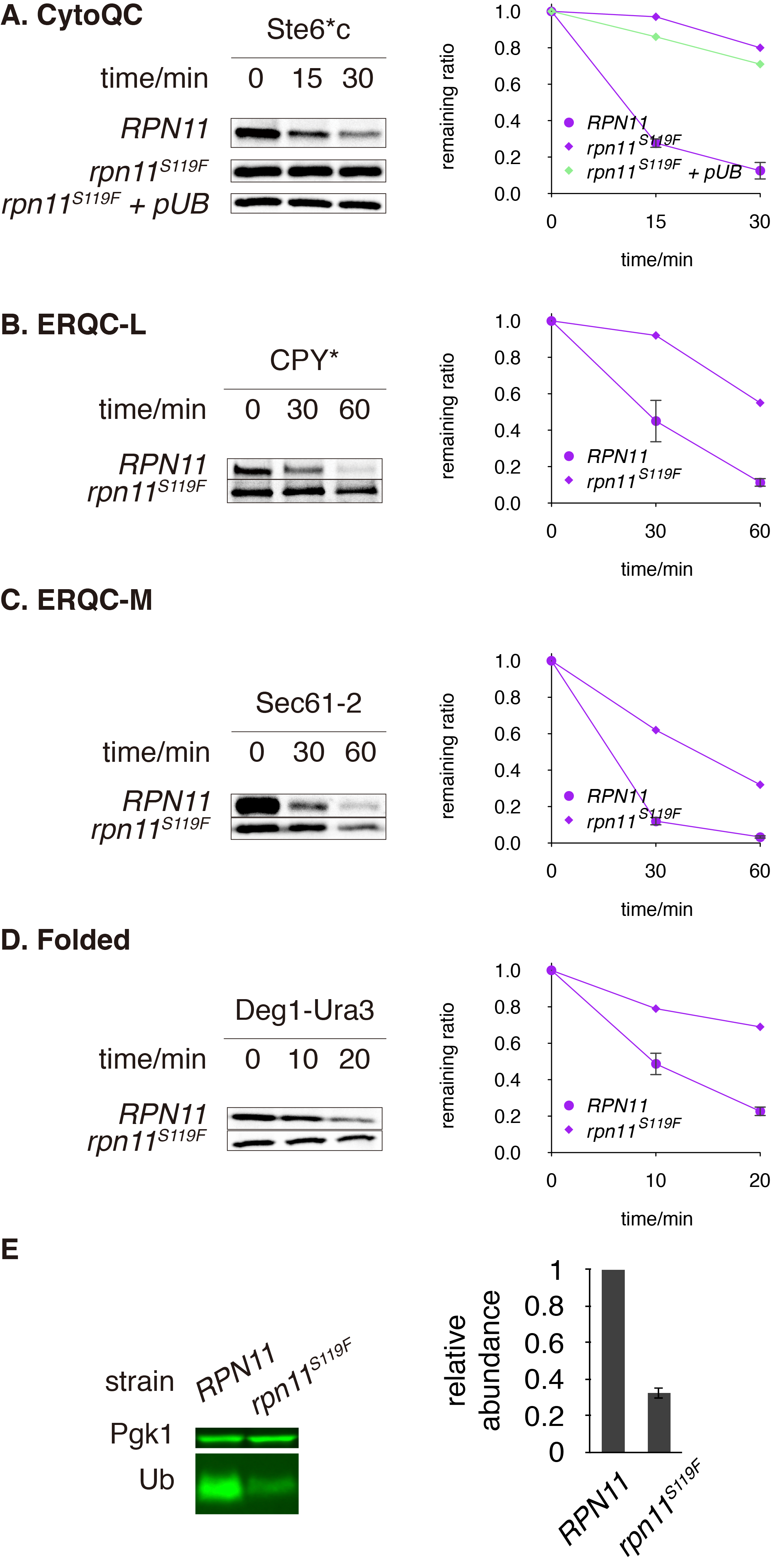


## Figure S4

## Figure S5


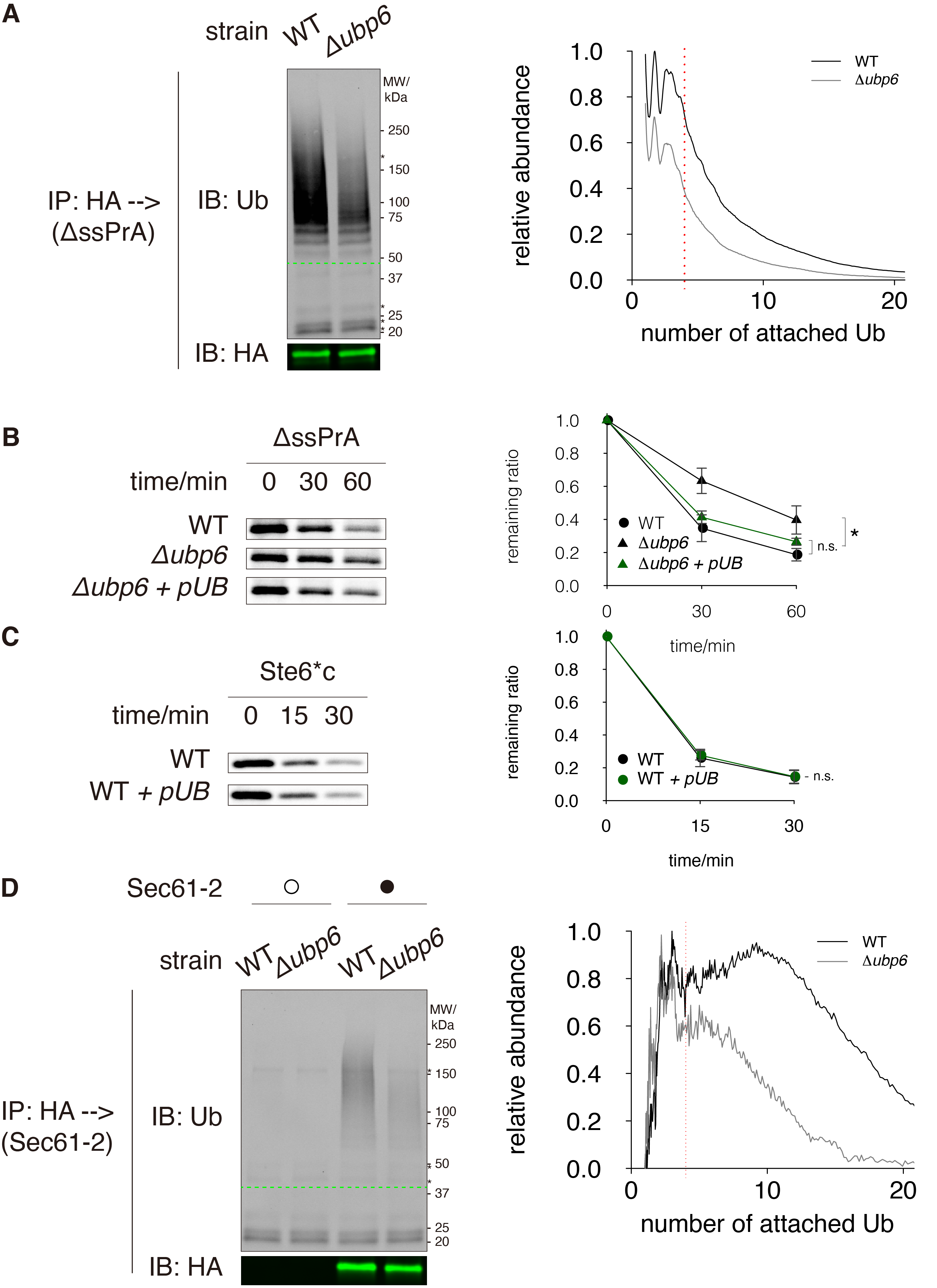


## Figure S6


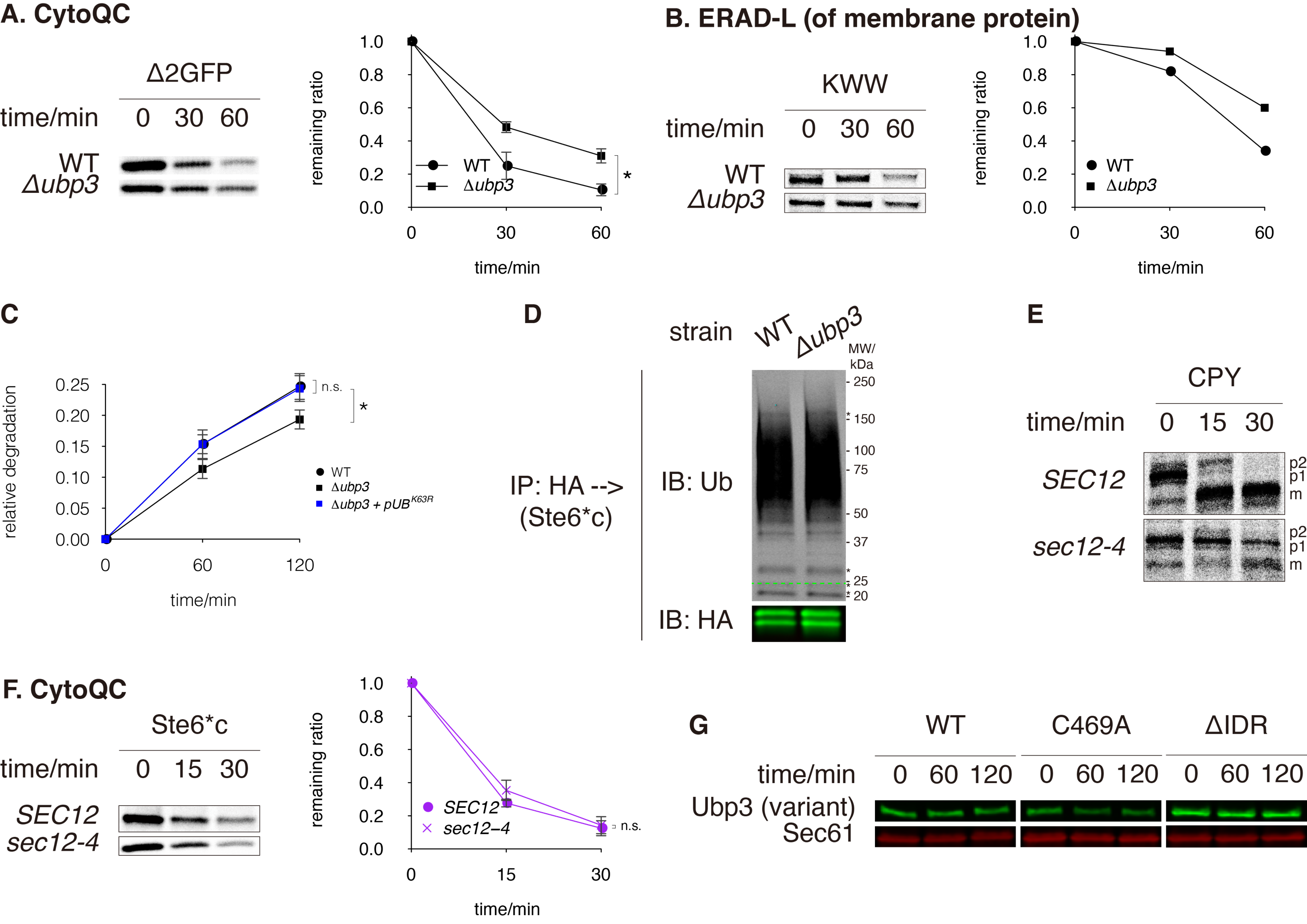


## Figure S7


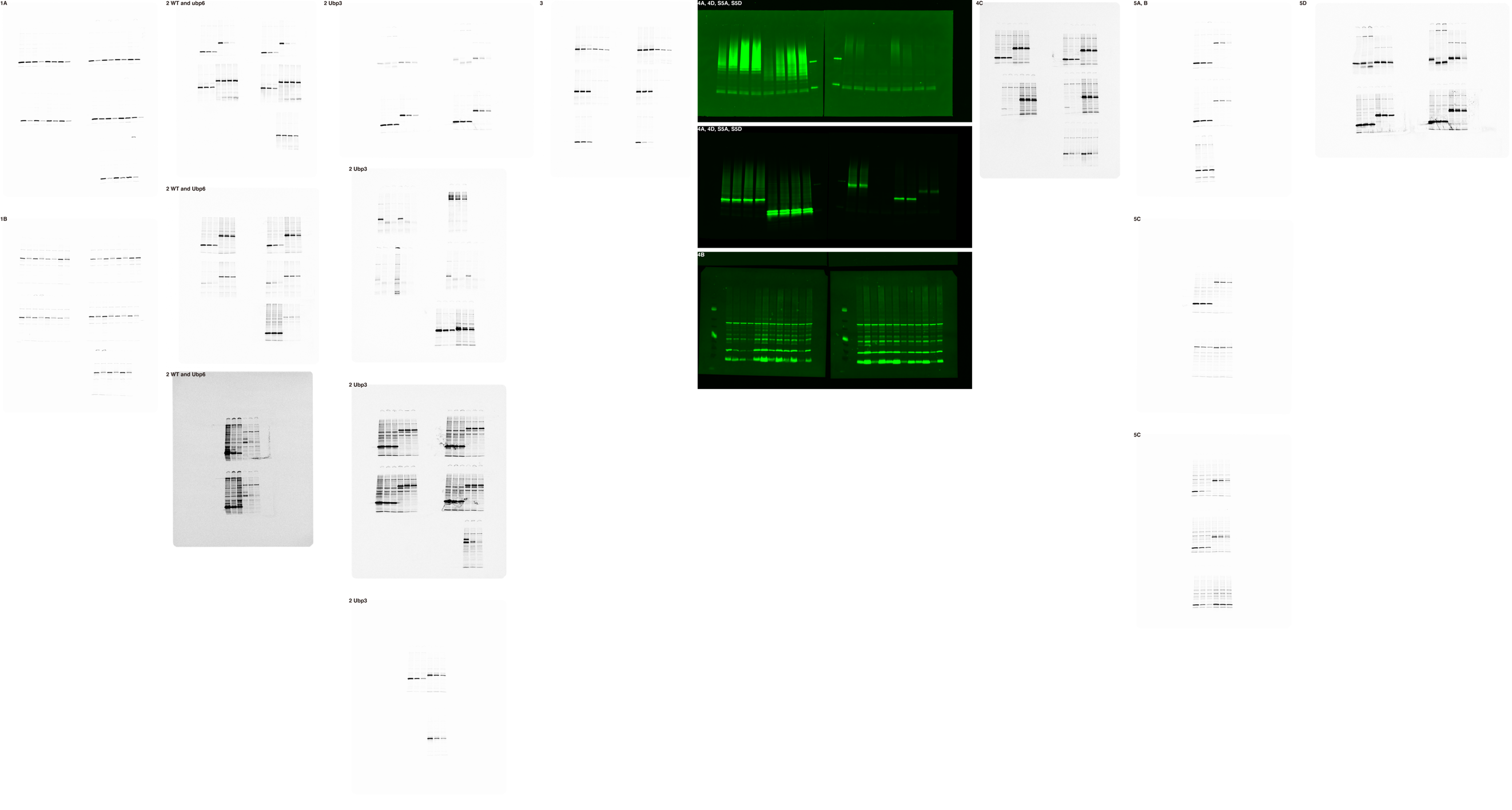


# Supplemental References

1. Prasad R, Kawaguchi S, Ng DT. A nucleus-based quality control mechanism for cytosolic proteins. Molecular biology of the cell. 2010;21(13):2117-27. Epub 2010/05/14. doi: 10.1091/mbc.E10-02-0111. PubMed PMID: 20462951; PubMed Central PMCID: PMCPmc2893977.

2. Prasad R, Kawaguchi S, Ng DT. Biosynthetic mode can determine the mechanism of protein quality control. Biochemical and biophysical research communications. 2012;425(3):689-95. Epub 2012/07/31. doi: 10.1016/j.bbrc.2012.07.080. PubMed PMID: 22842567.

3. Finger A, Knop M, Wolf DH. Analysis of two mutated vacuolar proteins reveals a degradation pathway in the endoplasmic reticulum or a related compartment of yeast. European journal of biochemistry. 1993;218(2):565-74. Epub 1993/12/01. PubMed PMID: 8269947.

4. Loayza D, Tam A, Schmidt WK, Michaelis S. Ste6p mutants defective in exit from the endoplasmic reticulum (ER) reveal aspects of an ER quality control pathway in Saccharomyces cerevisiae. Molecular biology of the cell. 1998;9(10):2767-84. Epub 1998/10/08. PubMed PMID: 9763443; PubMed Central PMCID: PMCPMC25553.

5. Biederer T, Volkwein C, Sommer T. Degradation of subunits of the Sec61p complex, an integral component of the ER membrane, by the ubiquitin-proteasome pathway. The EMBO journal. 1996;15(9):2069-76. Epub 1996/05/01. PubMed PMID: 8641272; PubMed Central PMCID: PMCPMC450128.

6. Vashist S, Ng DT. Misfolded proteins are sorted by a sequential checkpoint mechanism of ER quality control. The Journal of cell biology. 2004;165(1):41-52. Epub 2004/04/14. doi: 10.1083/jcb.200309132. PubMed PMID: 15078901; PubMed Central PMCID: PMCPmc2172089.

7. Gowda NK, Kandasamy G, Froehlich MS, Dohmen RJ, Andreasson C. Hsp70 nucleotide exchange factor Fes1 is essential for ubiquitin-dependent degradation of misfolded cytosolic proteins. Proceedings of the National Academy of Sciences of the United States of America. 2013;110(15):5975-80. Epub 2013/03/27. doi: 10.1073/pnas.1216778110. PubMed PMID: 23530227; PubMed Central PMCID: PMCPMC3625341.

8. Johnson PR, Swanson R, Rakhilina L, Hochstrasser M. Degradation signal masking by heterodimerization of MATalpha2 and MATa1 blocks their mutual destruction by the ubiquitin-proteasome pathway. Cell. 1998;94(2):217-27. Epub 1998/08/08. PubMed PMID: 9695950.

9. Abdel-Sater F, Jean C, Merhi A, Vissers S, André B. Amino acid signaling in yeast: activation of Ssy5 protease is associated with its phosphorylation-induced ubiquitylation. The Journal of biological chemistry. 2011;286(14):12006-15. Epub 2011/02/10. doi: 10.1074/jbc.M110.200592. PubMed PMID: 21310956.

10. El-Gebali S, Mistry J, Bateman A, Eddy SR, Luciani A, Potter SC, et al. The Pfam protein families database in 2019. Nucleic acids research. 2019;47(D1):D427-d32. Epub 2018/10/26. doi: 10.1093/nar/gky995. PubMed PMID: 30357350; PubMed Central PMCID: PMCPMC6324024.

11. Moore AD, Held A, Terrapon N, Weiner J, 3rd, Bornberg-Bauer E. DoMosaics: software for domain arrangement visualization and domain-centric analysis of proteins. Bioinformatics (Oxford, England). 2014;30(2):282-3. Epub 2013/11/14. doi: 10.1093/bioinformatics/btt640. PubMed PMID: 24222210.

12. Kosugi S, Hasebe M, Tomita M, Yanagawa H. Systematic identification of cell cycle-dependent yeast nucleocytoplasmic shuttling proteins by prediction of composite motifs. Proceedings of the National Academy of Sciences of the United States of America. 2009;106(25):10171-6. Epub 2009/06/13. doi: 10.1073/pnas.0900604106. PubMed PMID: 19520826; PubMed Central PMCID: PMCPMC2695404.
